# Hypertension in obese children is associated with vitamin D deficiency and serotonin dysregulation

**DOI:** 10.1186/s12887-022-03337-8

**Published:** 2022-05-17

**Authors:** Katarína Krivošíková, Zora Krivošíková, Ladislava Wsolová, Tomáš Seeman, Ľudmila Podracká

**Affiliations:** 1 Department of Pediatrics, National Institute of Children’s Diseases and Faculty of Medicine, Comenius University, Limbová 1, Bratislava, 831 01 Slovak Republic; 2 Department of Clinical and Experimental Pharmacotherapy, Faculty of Medicine, Slovak Medical University, Bratislava, Slovak Republic; 3 Department of Biophysics, Informatics and Biostatistics, Faculty of Public Health, Slovak Medical University, Bratislava, Slovak Republic; 4 Department of Pediatrics, 2nd Medical Faculty, Charles University Prague, Prague, Czech Republic; 5 Department of Pediatrics, Dr. von Hauner Children’s Hospital, University Hospital, LMU Munich, Munich, Germany

**Keywords:** Hypertension, Obesity, Serotonin, Vitamin D, Children

## Abstract

**Background:**

Obesity and hypertension represent serious health issues affecting the pediatric population with increasing prevalence. Hypovitaminosis D has been suggested to be associated with arterial hypertension. Serotonin by modulating nitric oxide synthase affect blood pressure regulation. The biological mechanism by which vitamin D specifically regulates serotonin synthesis was recently described. The aim of this paper is to determine the associations between vitamin D, serotonin, and blood pressure in obese children.

**Methods:**

One hundred and seventy-one children were enrolled in the prospective cross-sectional study. Two groups of children divided according to body mass index status to obese (BMI ≥95th percentile; *n* = 120) and non-obese (*n* = 51) were set. All children underwent office and ambulatory blood pressure monitoring and biochemical analysis of vitamin D and serotonin. Data on fasting glucose, insulin, HOMA, uric acid, and complete lipid profile were obtained in obese children.

**Results:**

Hypertension was found only in the group of obese children. Compared to the control group, obese children had lower vitamin D and serotonin, especially in winter. The vitamin D seasonality and BMI-SDS were shown as the most significant predictors of systolic blood pressure changes, while diastolic blood pressure was predicted mostly by insulin and serotonin. The presence of hypertension and high-normal blood pressure in obese children was most significantly affected by vitamin D deficiency and increased BMI-SDS.

**Conclusions:**

Dysregulation of vitamin D and serotonin can pose a risk of the onset and development of hypertension in obese children; therefore, their optimization together with reducing body weight may improve the long-term cardiovascular health of these children.

**Supplementary Information:**

The online version contains supplementary material available at 10.1186/s12887-022-03337-8.

## Introduction

Over the last decade, the incidence of pediatric hypertension has increased in parallel with obesity and its metabolic consequences. This trend also appears in the Slovak population of children [1]. Pediatric hypertension and especially in association with obesity represents the main predictor of adult hypertension and leads to increased cardiovascular mortality risk in adulthood [2, 3]. An analysis of data from over 57.915 overweight and obese children and adolescents revealed the prevalence of hypertension to be 20–40% [4].

The pathophysiology of hypertension in obese children appears to be complex and interdependent. It includes sympathetic nervous system activation by central mediation in the hypothalamus and local peripheral action, the effect of hyperleptinemia and hyperinsulinemia, endothelial dysfunction and oxidative stress, vascular damage as a consequence of inflammation, or vasoconstriction, and sodium and fluid retention through the renin-angiotensin system (RAS) activation [5–7]. Obesity also increases the overall cardiovascular risk with its other complications, such as type 2 diabetes mellitus, dyslipidemia, and left ventricular hypertrophy [8, 9].

Childhood obesity has been associated with low circulating serum concentrations of vitamin D [10], with vitamin D supplementation having a 30% lower response to the same dose of vitamin D in non-obese counterparts [11]. Vitamin D can affect cardiometabolic risk factors and plays an essential role in glucose homeostasis regulation, mechanisms of insulin secretion, and obesity-associated inflammation [12, 13]. Vitamin D deficiency has been suggested to be associated with arterial hypertension [14–16]. Impaired vitamin D receptor signaling leads to increased RAS activity, which then increases vascular stiffness, leading to impaired systolic and diastolic heart function [17]. The deficiency of vitamin D, as a negative endocrine regulator of renin biosynthesis, leads to elevated renin and angiotensin II production, consequently leading to high blood pressure (BP) and cardiac hypertrophy [18, 19]. With insufficient vitamin D concentrations, endothelial nitric oxide synthase (NOS) transcription is inefficiently regulated, and therefore reduced levels of NO can affect endothelium-dependent vascular relaxation, thus increasing vascular contractile activity [20].

In 2014, evidence on biological mechanisms by which vitamin D specifically regulates tissue-specific serotonin (5-HT) synthesis has been published [21]. There is in vitro and in vivo evidence that 5-HT causes a long-term decrease in BP that is dependent on NOS stimulation by serotonin in vascular endothelial cells and neurons [22]. Serotonin released from activated platelets via 5-HT_2_ receptors directly induces vasoconstriction, and indirectly contributes to vasoconstriction by enhancing the contractile ability of other vasoactive substances such as angiotensin II, histamine, etc. [23]. In contrast, serotonin via 5-HT_1_ receptors stimulates the release of NO from the endothelium, leading to the relaxation of vascular smooth muscle cells. NO also acts as an antiregulatory mechanism against the natural vasoconstrictive effects of serotonin [24].

The present study aimed to confirm the hypothesis that obese Slovak children have vitamin D deficiency and hypertension; and investigate the relationship between vitamin D and serotonin in association to blood pressure in obesity.

## Methods

### Study population

Of 181 children in the Department of Pediatrics of the National Institute of Children’s Diseases during 2018–2019, 120 obese and 51 lean children were enrolled in the prospective cross-sectional study. Due to the lack of reference values for mean day-time systolic, diastolic, and mean arterial BP relative to height less than 120 cm, ten children (1 obese child and 9 lean children) were excluded from this analysis. Gender was evenly distributed with 89 females and 82 males. The mean age for this cohort was 12.9 years +/− 3.0 years.

Subjects were divided into two groups according to body mass index (BMI) status, a group of children with obesity (*n* = 120) and without obesity (*n*  =51), respectively. Obesity was defined as BMI ≥95th percentile growth reference for Slovak children [25, 26] aged 0–18 years. The inclusion criteria for the obese children’s group comprised the following: obesity lasting more than 4 years; no history of underlying diseases or family history of diabetes; no medical treatment for weight control in the previous 12 months; no vitamin D supplementation for at least 2 months prior to the study. Blood tests were performed evenly throughout the year, independently of the year season. The exclusion criteria included monogenic, syndromological or secondary cause of obesity, diabetes mellitus and secondary hypertension.

All obese subjects initially referred either from a general practitioner’s office or a district endocrinologist were hospitalized at the Department of Pediatrics of the National Institute of Children’s Diseases during 2018–2019 in regard to investigating the causes of obesity, its health impact, and possible complications.

Fifty-one age- and sex-matched healthy lean peers without hypertension, known chronic or acute disease were recruited at the outpatient clinic during the check-up after overcoming a disease unrelated to obesity or hypertension during the same time period.

Anthropometric measurements were assessed according to standardized protocols. Body weight was measured with an electronic tensiometer scale (Tonava TH200, Tonava, Prague, Czech Republic) with a precision of 0.1 kg; body height was assessed using a built-in stadiometer to the nearest 0.5 cm.

Legal guardians of all children provided written informed consent before inclusion. The study was approved by the Ethical Board of National Institute of Children’s Diseases, Bratislava, Slovakia no. EK:05/2018 and was conducted in accord with the Helsinki Declaration.

### Office blood pressure measurement

Office BP was measured according to current guidelines [27] during one visit. The BP measurement was performed with the automatic oscillometer Omron HBP-1300 (Omron Healthcare, IL, USA) validated for children [28]. Subsequently, the values were converted to SDS based on pediatric BP normative values [29]. High-normal office blood pressure (HNBP) was defined as office BP ≥90th percentile but <95th percentile; and office hypertension defined as BP ≥95th percentile (equivalent to 1.645 SDS) for age, height, and gender according to normative tables published in the Fourth Report [27]. See Supplementary 1 for further details.

### 24-hour ambulatory blood pressure monitoring

24-hour ambulatory BP monitoring (ABPM) was performed in all obese patients to confirm hypertension in accordance with current guidelines [27]. Patients underwent ABPM using the SpaceLabs 90,217 oscillometric device (Spacelabs Healthcare, Hertford, UK). High-normal ambulatory blood pressure was defined as either daytime and/or nighttime systolic and/or diastolic BP means ≥90th percentile (equivalent to the 1.28 SDS) but <95th percentile; and ambulatory hypertension according to ABPM was defined as either daytime and/or nighttime systolic and/or diastolic BP means ≥95th percentile (equivalent to the 1.645 SDS) [27, 30]. Normative BP data were derived from gender- and age-matched children of the same height centiles. See Supplementary 1 for further details.

### Obesity classification

Obesity was defined as a BMI equal or greater than the age- and sex-appropriate 95th percentile (equivalent to the 1.65 SDS). BMI was calculated as weight in kilograms divided by height in meters squared. To compare BMI values across different ages and by gender, the BMI-SDS was calculated according to the BMI growth reference for Slovak children aged 0–18 years [25, 26].

### Definition of vitamin D insufficiency and deficiency

According to the Endocrine Society vitamin D status was defined as followed: 25(OH) D levels ≥30 ng/mL (≥75 nmol/L) were set as normal values, vitamin D insufficiency as a 25(OH) D levels of 20–30 ng/mL (50–75 nmol/L) and vitamin D deficiency as a 25(OH) D level < 20 ng/mL (< 50 nmol/L) [31].

### Biochemical analyses

Blood was collected from the antecubital vein after fasting overnight. In the central laboratory, serum total cholesterol (T-Chol), LDL-cholesterol (LDL-C), HDL-cholesterol (HDL-C), triacylglycerols (TAG), fasting glucose (Glu), uric acid on a Cobas 501 analyzer (Roche, CA, USA), insulin on a Cobas E401 analyzer (Roche, CA, USA) and vitamin D_3_ (25(OH)D) on a Vitros 5600 analyzer (Vitros, Johnson & Johnson, Rochester, NY, USA) were analyzed using standard laboratory methods. LDL-C was calculated according to Friedewald formula [32]. The HOMA index (Homeostatic Model Assessment) was calculated according to the formula: [serum insulin concentration (ng/mL) × serum glucose concentration (mmol/L)] /22.5 [33]. The rest of the serum was stored at − 70 °C until further analysis. Serum serotonin concentrations were measured by ELISA kit (Serotonin ELISA, DRG Instruments GmbH, Germany).

### Statistical analysis

Statistical analysis was performed using SPSS software, version 23.0 (IBM Corp., NY, USA). Categorical variables were reported as counts and percentages, and continuous variables as arithmetic mean (X̅) ± standard deviation (SD). Shapiro-Wilk test was used to check continuous variables for normality. Differences between groups in quantitative variables were evaluated by two sample t test or the Mann-Whitney U test, as appropriate. Proportions were compared by the *χ*^2^ test. *P* values < 0.05 were considered statistically significant. Binary stepwise logistic regression analysis was used to estimate OR and 95% CI for the prediction of HNBP and hypertension in obese children. Decision trees were employed to study the impact of independent variables on SBP-SDS, DBP-SDS, and the prevalence of hypertension, respectively.

## Results

General characteristics of children who participated in this study are summarized in Table 1. The ratio of boys and girls and their age did not differ statistically in individual groups. The control group had significantly lower BMI-SDS (*p* < 0.001), day SBP-SDS (*p* < 0.001), concentrations of 25(OH) D (*p* < 0.001) and serotonin (*p* = 0.033). Obese girls did not differ statistically significantly from obese boys in anthropometric parameters, BP values, as well as followed metabolic hormones (Supplementary 2).

**Table 1:** Demographic and laboratory data for the study group

	Obese	Normal weight	***p*** value
**Number (male/female)**	120 (57/63)	51 (25/26)	0.856
**Age (years)**	13.0 ± 2.9	12.6 ± 3.1	0.476
**Height (m)**	1.63 ± 0.14	1.56 ± 0.15	0.068
**Height-SDS**	0.61 ± 1.03	0.07 ± 0.90	**0.001**
**Weight (kg)**	89.90 ± 23.46	49.79 ± 14.66	**0.000**
**BMI (m**^**2**^**/kg)**	33.52 ± 6.43	19.78 ± 2.88	**0.000**
**BMI-SDS**	5.61 ± 2.44	0.31 ± 0.90	**0.000**
**Day SBP (mmHg)**	121.73 ± 11.63	110.35 ± 8.23	**0.000**
**Day DBP (mmHg)**	71.59 ± 8.62	68.02 ± 7.57	**0.035**
**Day SBP-SDS**	0.38 ± 1.41	−0.87 ± 1.00	**0.000**
**Day DBP-SDS**	−0.15 ± 1.48	− 0.67 ± 1.18	0.064
**25(OH) D (ng/mL)**	27.75 ± 8.58	34.24 ± 9.31	**0.000**
**Serotonin (ng/mL)**	162.75 ± 79.63	241.53 ± 109.06	**0.033**
**Glu (mmol/L)**	4.04 ± 0.86	4.88 ± 0.71	**0.003**
**Insulin (mU/L)**	28.64 ± 16.90	21.18 ± 8.74	0.291
**HOMA**	5.55 ± 3.93	4.55 ± 2.27	0.499

High-normal BP or hypertension (HNBP/HT) was found only in the group of obese children. Table 2 shows the distribution of children with normal BP, HNBP, systolic, systolic-diastolic, and diastolic hypertension. Slight, but statistically significant higher prevalence of HNBP/HT was found in boys (*p* < 0.040). In individual forms of hypertension, boys suffer more from HNBP and diastolic hypertension and girls from systolic hypertension.

**Table 2:** Classification of blood pressure in obese children

BP classification:	Obese boys (***n*** = 57)	Obese girls (***n*** = 63)	***p*** value
**Normal BP**	40 (70.2%)	46 (73.0%)	
**HNBP/HT**	17 (29.8%)	17 (27.0%)	**0.04**
High-normal blood pressure	5 (8.8%)	1 (1.6%)	
Systolic hypertension	3 (5.3%)	10 (15.9%)	
Diastolic hypertension	7 (12.3%)	2 (3.2%)	
Systolic-diastolic hypertension	2 (3.5%)	4 (6.3%)	
**White-coat hypertension**	25 (43,9%)	15 (23.8%)	
**Masked hypertension**	2 (3.5%)	6 (9.5%)	

*BP* blood pressure, *HNBP/HT* high-normal blood pressure/hypertension

When evaluating vitamin D status, a significant difference was found between obese and lean children (*p* < 0.002; Table 3). Only 37.1% of the obese children had optimal values of 25(OH) D (≥30 ng/mL) in contrast to 68% of lean children. The risk of vitamin D deficiency was three times higher in obese children compared to lean children (*p* < 0.002; OR: 2.996).

**Table 3:** Vitamin D status in children with and without obesity

25(OH) D (ng/mL)	Obese (***n*** = 116; 100%)	Normal weight (***n*** = 47; 100%)	***p*** value; OR
**≥30 (optimal)**	**43 (37.1%)**	**30 (63.8%)**	**0.002;**
**< 30 (deficiency)**	**73 (62.9%)**	**17 (36.2%)**	**2.996**
≥20 < 30 ng/mL (mild)	54 (46.6%)	16 (22.9%)	
≥10 <20 ng/mL (moderate)	19 (16.4%)	1 (2.1%)	
< 10 ng/mL (severe)	0 (0%)	0 (0%)	

*OR* odds ratio

Mean serum concentrations of 25(OH) D and serotonin during the whole year and in individual seasons are shown in Table 4. 25(OH) D and serotonin levels were significantly higher in lean children if compared to obese children throughout the whole year (*p* < 0.000; *p* < 0.033; respectively) as well as in the summer-autumn season (*p* < 0.001; *p* < 0.000; respectively). In the winter-spring season, the concentrations were significantly higher only for 25(OH) D (*p* < 0.019).

**Table 4:** 25(OH) D and Serotonin in individual groups according to year seasons

Whole year	Obese (***n***)	Normal weight (***n***)	***p*** value
**25(OH) D (ng/mL)**	27.75 ± 8.58 (116)	34.24 ± 9.31 (47)	**0.000**
**Serotonin (ng/mL)**	162.75 ± 79.63 (119)	241.53 ± 109.06 (45)	**0.033**
**Winter-spring**			
**25(OH) D (ng/mL)**	27.17 ± 7.56 (42)	34.15 ± 6.66 (8)	**0.019**
**Serotonin (ng/mL)**	154.49 ± 46.98 (42)	157.00 ± 58.62 (8)	0.895
**Summer-autumn**			
**25(OH) D (ng/mL)**	28.08 ± 9.14 (74)	34.26 ± 9.84 (39)	**0.001**
**Serotonin (ng/mL)**	167.26 ± 92.71 (77)	259.81 ± 109.26 (37)	**0.000**

**Fig. 1:**
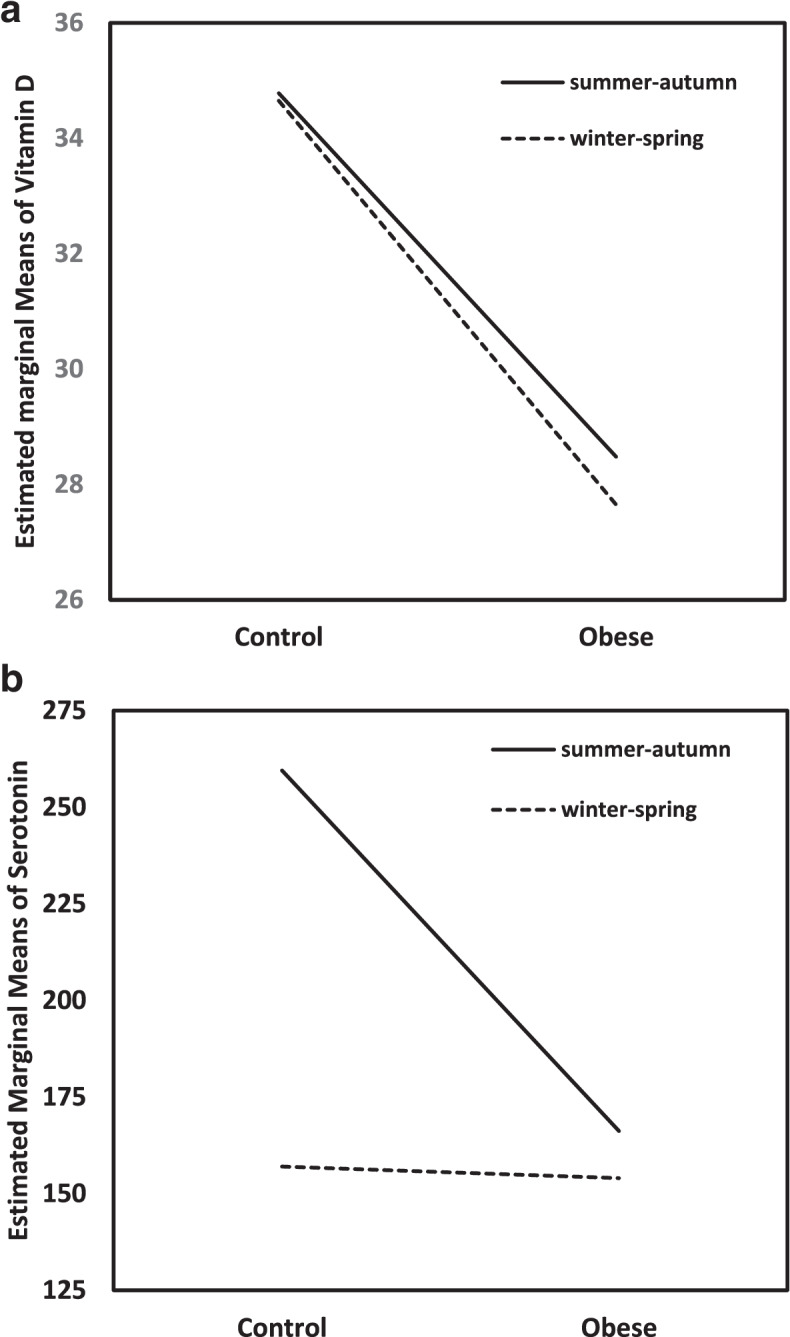
Vitamin D and Serotonin levels depending on Vitamin D seasonality and study group. **a** General linear model for 25(OH) D as a response variable and vitamin D seasonality and obesity as predictors. **b** General linear model for serotonin as a response variable and vitamin D seasonality and obesity as predictors

However, the distribution of children according to both studied groups and particular seasons was significantly different (*p* < 0.023). Therefore, the general linear models (GLMs) were constructed with 25(OH) D (Fig. 1a) and serotonin (Fig. 1b) as dependent variables and vitamin D seasonality and distribution of obese and nonobese children as fixed factors. GLM confirmed significantly higher 25(OH) D concentrations (*p* < 0.001) in the control group in both the summer-autumn season and the winter-spring season in comparison to the obese group independently from the children’s distribution in studied groups and particular seasons (*p* < 0.834). In contrast, we found significant interactions between serotonin (*p* < 0.018) and seasonality in the control group. In the group of obese children, no effect of season on serotonin was found.

Multivariable stepwise regression analysis was performed to assess the predictive effect of risk factors on HNBP/HT (Table 5). The most predictive risk factors were found to be BMI-SDS (OR = 1.480, 95% CI: 1.152–1.900), T-Chol (OR = 2.285, 95% CI: 1.309–3.991) and vitamin D seasonality (OR = 3.975, 95% CI: 1.246–12.686). In children with obesity, HNBP/HT was 1.48 times more frequent in comparison to lean children. Similarly, higher levels of T-Chol and the winter-spring season were responsible for 2.285 and 3.975 times more frequent prevalence of HNBP/HT.

**Table 5:** Logistic Regression Analysis of Risk factors of HNBP/HT in obese children

Included variables	ß	***p*** value	OR (95% CI)	Excluded variables
**BMI-SDS**	0.392	0.002	1.480 (1.152–1.900)	age, gender, 25(OH) D, vitamin D deficiency, serotonin, Glu, LDL-C, HDL-C, TAG, insulin, HOMA
**T-Chol**	0.827	0.004	2.285 (1.309–3.991)	
**Vitamin D Seasonality**	1.380	0.020	3.975 (1.246–12.686)	

*β* standardized coefficients, *OR* Odds Ratio, *95% CI* Confidential Interval

**Fig. 2:**
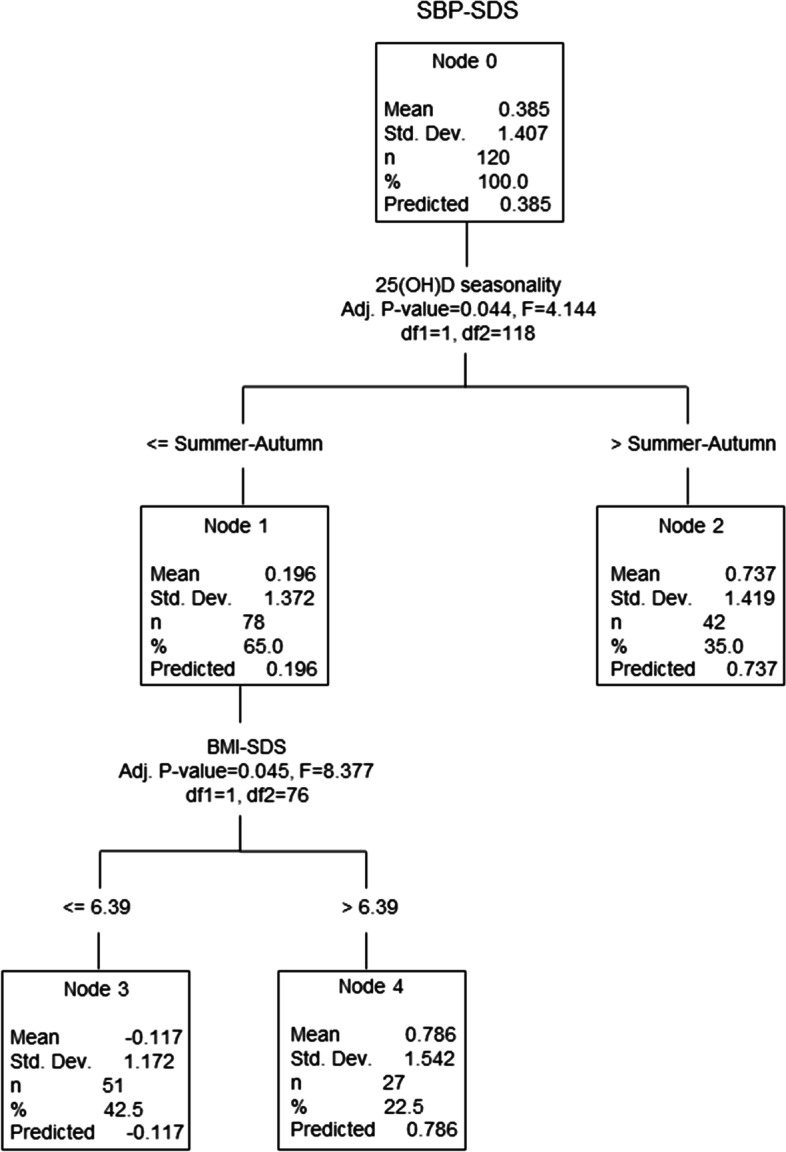
Multivariable analysis of the relationship between SBP-SDS and studied variables. SBP-SDS: systolic blood pressure standard deviation score, BMI-SDS: body mass index standard deviation score

The impact of the particular combination of variables (age, gender, BMI-SDS, vitamin D deficiency, vitamin D seasonality, 25(OH) D, serotonin, T-Chol, LDL-C, HDL-C, TAG, Glu, insulin, HOMA) on SBP-SDS, DBP-SDS and the BP classification was further investigated using the SPSS decision tree. Figure 2 illustrates the vitamin D seasonality and BMI-SDS as the best predictors found for SBP-SDS. During the summer-autumn season, SBP-SDS was significantly lower compared to SBP-SDS measured in the winter-spring season (0.196 vs. 0.737; *p* < 0.044). Furthermore, the decision tree continued dividing children measured in the summer-autumn season by BMI-SDS. Children with BMI-SDS ⋜6.39 had significantly lower SBP-SDS (*p* < 0.045) in comparison to children with BMI-SDS > 6.39.

**Fig. 3:**
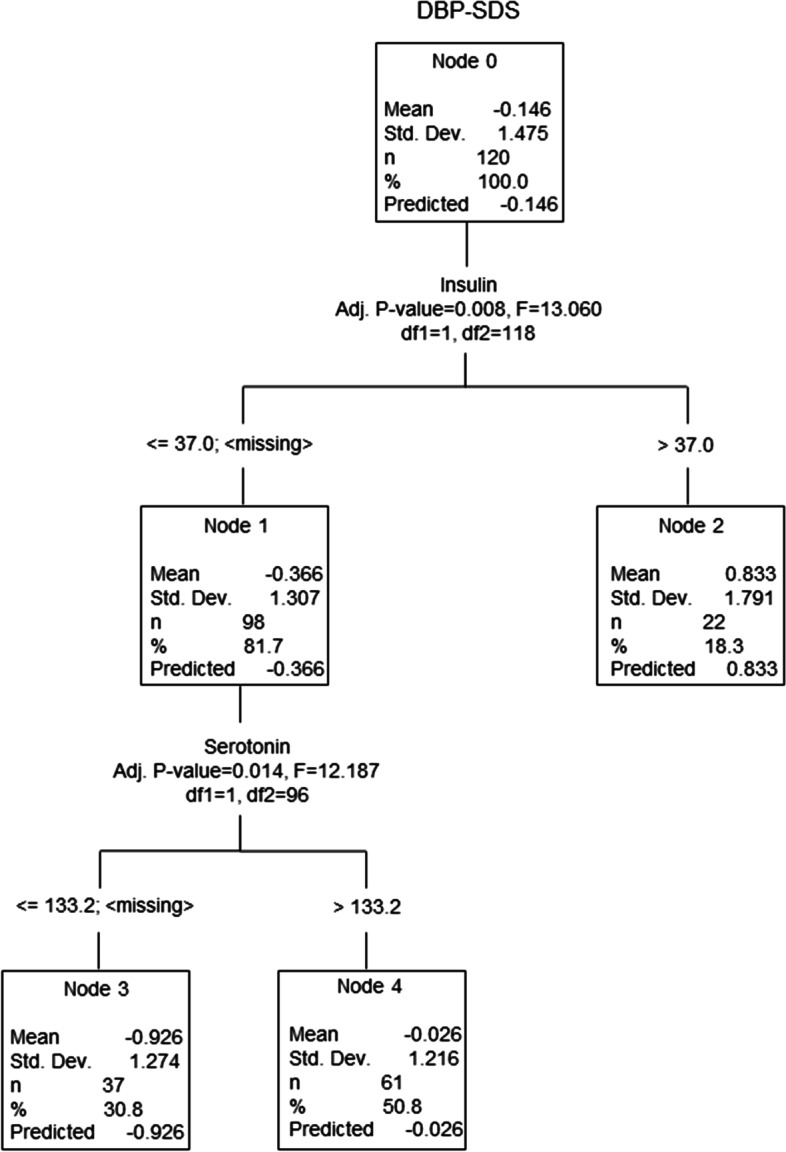
Multivariable analysis of the relationship between DBP-SDS and studied variables. DBP-SDS: diastolic blood pressure standard deviation score

The best predictors of DBP-SDS in the group of children with obesity are illustrated in Fig. 3. Children with insulin ⋜37.0 (first to third quartile) had significantly lower DBP-SDS (− 0.366) than children with insulin > 37.0 (0.833; *p* < 0.008). In the next step, children with lower insulin levels were divided according to serotonin levels. Children with serotonin ⋜133.2 had significantly lower DBP-SDS (− 0.926) compared to children with serotonin > 133.2 (− 0.026; *p* < 0.014).

**Fig. 4:**
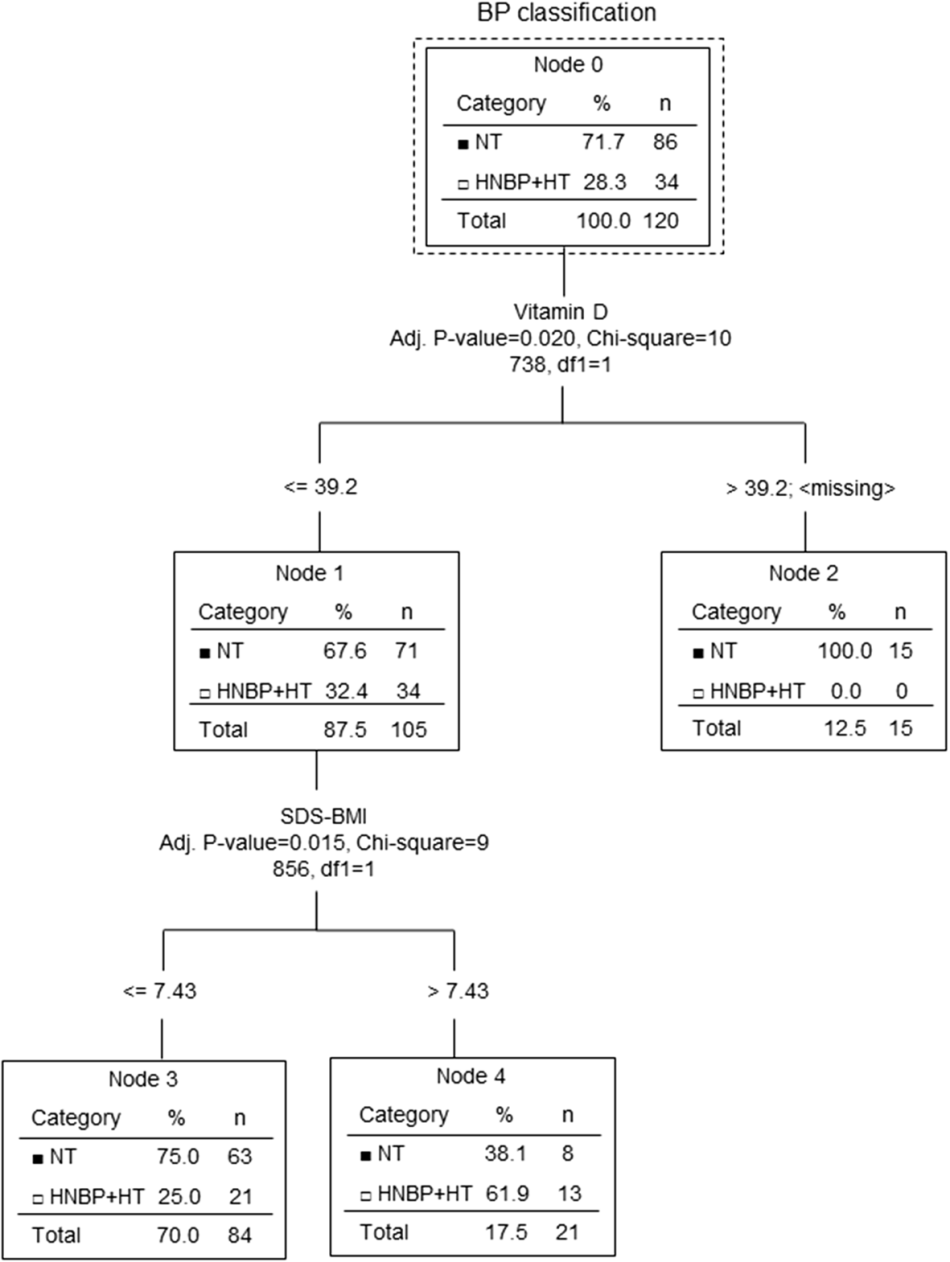
Multivariable analysis of the relationship between the prevalence of hypertension and studied variables. BP: blood pressure; SDS-BMI: body mass index standard deviation score

The decision tree selected 25(OH) D as a main determinant of hypertension prevalence in the studied group of children with obesity (Fig. 4). Children with 25(OH) D levels > 39.2 had no HNBPHT compared to children with 25(OH) D levels ⋜39.2 ng/mL, where the distribution was 32.4% of HNBP/HT vs. 67.6% of NT (*p* < 0.02). Among the children with lower 25(OH) D levels, subjects with BMI-SDS ⋜7.43 had significantly higher prevalence of normotension (NT) (75.0%) vs. HNBP/HT (25.0%) in comparison to those with BMI-SDS > 7.43 (38.1% vs. 61.9%; *p* < 0.015).

## Discussion

The global prevalence of hypertension due to obesity pandemic in children and adolescents has increased persistently [34–36]. In Slovakia, there are only limited data on obese children in association with other comorbidities. In 2012, Babinska et al. [1] reported that only 24% of Slovak children with obesity had normal BP, while 25% suffered from HNBP and 51% from hypertension. In contrast, in our study, 71.7% of obese children had normal BP, 5% had HNBP, 23.3% had any form of hypertension. Our results are in concordance with other recent reports [4, 37]. The importance of the influence of BMI on the onset and development of hypertension is also confirmed by a Danish study, in which systolic BP was significantly reduced by reducing BMI SDS by more than 0.25 [38]. Children with normal weight in our study did not have HNBP or hypertension, however, there are studies reporting also normal-weight children to be hypertensive [39, 40]. When comparing obese boys with girls, we found that boys suffer significantly more often from any type of hypertension. Results from similar studies are inconsistent [4, 37, 41–43]. The mechanisms by which biological sex contributes to this difference remain not fully clarified at present; nevertheless, several intriguing mechanistic candidates have been proposed ranging from different concentrations of sex hormones, utilization of fat stores to the effect of X or Y chromosome themselves [44–46]. Increasing secretion of sex hormones during puberty leads to changes in body composition, fuel metabolism, BP, lipid levels and decreased insulin sensitivity [47–53]. A positive relationship has previously been observed between the amount of adipose tissue and BP. Obesity represents a well-established risk factor for development of hypertension in both girls and boys [7]. Body fat relates positively to insulin resistance that, in turn, has been associated with the development of hypertension [54, 55]. Since we did not find significant sex differences in cardiometabolic parameters or BP values in the present study, our objective was to identify the most significant predictors of the development of hypertension. Total cholesterol and BMI-SDS predicted HNBP and hypertension most significantly in the stepwise multivariable regression analysis, with a notable magnitude of the effect size. These were also identified to be the most important risk factors for the development of systolic, and altogether with insulin also of diastolic hypertension. This possible predominant role of insulin in influencing DBP values is in agreement with a study conducted by Marcovecchio et al. [55] in which mean 24-h and daytime DBP were significantly related to insulin resistance. Hyperinsulinemia has been suggested to lead to increased sodium absorption in the kidneys and sympathetic nervous system activity [56]. Activation of RAS with vascular endothelial dysfunction, caused by various inflammatory cytokines, especially those such as interleukin-6, resistin, tumor necrosis factor-α, lower level of NO observed in the context of insulin resistance, have been reported to contribute to the development of hypertension [56, 57].

According to our data, serotonin concentrations were lower in obese children with significant differences during year seasons. In the winter-spring season, lower serotonin concentrations were observed in both study groups and were very similar. In summer-autumn season, serotonin levels in obese children remained unchanged, but raised significantly in lean group. Partially, hypovitaminosis D could contribute to lower serotonin levels in obese children since vitamin D is known to regulate serotonin synthesis [21]. However, this does not explain low serotonin in lean children who have optimal vitamin D levels. Regarding the serotonin levels in obesity, the evidence described in the literature is controversial. The results of several human studies agree with our results and reported a negative association between serotonin concentrations and weight or BMI [58–60]. The explanation could be that circulating serotonin interacts with leptin in adipose tissue and increases the feeling of satiety [59]; therefore, it could be considered protective against obesity. Conversely, animal studies showed high levels of serotonin to be related to obesity [61, 62]. In accordance with these studies, high-fat diet promotes the overexpression of TPH_1_, which increases the serotonin levels, consequently promoting gluconeogenesis and lipogenesis, leading to obesity. The reason for these discrepancies could be explained by several reasons: usage of different determination methods (fluorometry, ELISA, HPLC, etc.) [58]; different diets prior to measurements (high-fat or carbohydrate-rich diet associated with increase in serotonin levels [61, 62] in contrast to low-calorie diet leading to decrease in serotonin [60]); comparison of different types of cohorts. In connection to BP, surprisingly, we have found serotonin has been shown to be an important determinant of diastole in obese children without hypertension who had insulin concentrations corresponding to the first to third quartiles. According to recent publications, serotonin can have a dual effect on BP depending on which receptor it acts on. By binding to 5-HT_2_ receptors, serotonin induces vasoconstriction, while through 5-HT_1_ receptors it causes vasodilation via stimulation of NO release from vascular endothelium [23, 24]. Vitamin D also positively regulates NO synthesis [20]. We can speculate that obese children with lower serotonin and vitamin D deficiency have reduced NO production and thus a higher risk of developing hypertension. Since a similarly designed study on the relationship between serotonin and BP in normotensive children with obesity has not yet been published, it is difficult to interpret our results correctly.

Vitamin D itself may be linked to the regulation of BP. As described in the introduction, vitamin D deficiency is involved in the pathomechanism of the onset and development of high BP, which is confirmed by the result of our study evaluating vitamin D as the most important determinant of hypertension in obese children. Likewise, outcomes of several other studies supported the inverse relationship between vitamin D and BP [14, 15, 18, 63]. Simultaneously, the randomized clinical trial by Rajakumar et al. [64] showed that correction of vitamin D deficiency in overweight and obese children by vitamin D supplementation resulted in reduction in BP.

This study had some limitations. In this work, we used the classification of vitamin D deficiency by Holick et al. [31] according to which the serum 25(OH) D value of 20 ng/ml is accepted as the deficiency threshold in adults. However, the cut-off concentration of 25(OH) D deficiency remains to be established in various pediatric age groups. Furthermore, recruitment and sampling were done throughout the year. Serum vitamin D levels are seasonal and higher concentrations in summer may not correspond to winter levels. In our study, we partially solved this by adjusting the multivariable analysis for the age of the monitored children and for the season. Finally, the number of subjects in both groups, obese and non-obese, was not proportionally equal if divided into subgroups according to seasons. Therefore, we used the general linear models with 25(OH) D and serotonin as dependent variables and vitamin D seasonality and distribution of obese and nonobese children as fixed factors. The general linear model confirmed a significantly higher concentration of 25(OH) D, but not serotonin, in the control group in both the summer-autumn season and the winter-spring season compared to the obese group independently of the subjects’ distribution in the studied groups and particular seasons.

To conclude, we have found that obesity in children is associated with decreased serotonin concentrations, as well as vitamin D. In particular, vitamin D deficiency and low serum serotonin have shown to be significant risk factors of arterial hypertension in obese children. On the other side, this study cannot specify the exact mechanisms by which serotonin and vitamin D are involved in the pathogenesis of pediatric hypertension and future research on this topic is needed. However, our findings suggest that dysregulation of these metabolic hormones can pose a risk of the onset and development of hypertension in obese children; therefore, their optimization through its potential beneficial effects on blood pressure may have a primary preventive role in improving the long-term cardiovascular health of these children.

## Supplementary Information

**Additional file 1.**

## Data Availability

The datasets generated and/or analysed during the current study are not publicly available due to the data protection and privacy of the patients hospitalized at the National Institute of Children’s Diseases in Bratislava, Slovakia, but are available from the corresponding author on reasonable request.

## Abbreviations

25(OH)D: Vitamin D
5-HT: Serotonin
ABPM: Ambulatory blood pressure monitoring
BMI: Body mass index
BP: Blood pressure
DBP: Diastolic blood pressure
ELISA: Enzyme-linked immunosorbent assay
GLMs: General linear models
Glu: Fasting glucose
HDL-C: HDL-cholesterol
HNBP: High-normal blood pressure
HOMA: Homeostatic model assessment for insulin resistance
HT: Hypertension
HPLC: High-performance liquid chromatography
LDL-C: LDL-cholesterol
NOS: Nitric oxide synthase
OR: Odds ratio
RAS: Renin-angiotensin system
SBP: Systolic blood pressure
SDS: Standard deviation score
TAG: Triacylglycerols
T-chol: Serum total cholesterol
TPH: Tryptophan hydroxylase
