# Supplementary material for: Hypertension in obese children is associated with vitamin D deficiency and serotonin dysregulation
Source: BMC Pediatr. 2022 May 17;22:289. doi: 10.1186/s12887-022-03337-8 (PMC9112480; doi:10.1186/s12887-022-03337-8)
Supplement: Supplementary file 1 — Additional file 1. [file 12887_2022_3337_MOESM1_ESM.docx]

**SUPPLEMENTARY 1**

**Office blood pressure measurement**

Office blood pressure (BP) was measured by a trained nurse according to current guidelines (1) during one visit. After 10 minutes of rest, the blood pressure measurement was performed with the automatic oscillometer Omron HBP-1300 (Omron Healthcare, IL, USA) validated for children (2). Three consecutive measurements were taken at 5 minutes intervals. The measurements were performed in the sitting position on the right arm using an appropriate cuff size of approximately 40% of the mid-arm circumference. The average values of both systolic (SBP) and diastolic blood pressure (DBP) from the 2^nd^ and 3^rd^ measurements were determined. The values were subsequently converted into SDS based on the pediatric blood pressure normative values (3). Normal-high blood pressure was defined as systolic and/or diastolic BP ≥ 90^th^ percentile but <95^th^ percentile for age, sex, and height according to normative tables published in the Fourth Report. Office hypertension was defined as either systolic and/or diastolic blood pressure values ≥95^th^ percentile (equivalent to the 1.645 SDS) for age, height, and gender (1).

**24-hour ambulatory blood pressure monitoring**

24-hour ambulatory blood pressure monitoring (ABPM) was performed in all obese patients to confirm hypertension in accordance with current guidelines (1). Patients underwent ABPM using a SpaceLabs 90217 oscillometric device (Spacelabs Healthcare, Hertford, UK). An appropriate cuff size of approximately 40% of the mid-arm circumference was chosen and attached to the non-dominant arm with free movement allowance during the day to reduce errors during measurements due to movement artifacts. All participants were instructed to keep a diary of events and daily activities over a 24-hour monitored period. Blood pressure was measured every 20 minutes during daytime (5 am–10 pm) and every 30 minutes during night-time (10 pm–5 am). Actual recordings were edited accordingly to real periods of sleep and wakefulness. A minimum of 40 ABPM recordings with at least 8 recordings during the night were accepted for the analysis. SpaceLabs Healthcare software (2010, USA) was used for the analysis of the ABPM results. Data of systolic, diastolic blood pressure and mean arterial pressure (MAP) measured over 24-hour, daytime and night-time periods were obtained and analyzed. The average absolute values of MAP, SBP, and DBP in all time periods were subsequently converted into SDS values using the reference data from a multicenter ABPM study (4).

Ambulatory hypertension according to ABPM was defined as either daytime and/or nighttime systolic and/or diastolic blood pressure means ≥95^th^ percentile (equivalent to the 1.645 SDS (1, 4). Normative blood pressure data were derived from gender - and age-matched children of the same height centiles. Normal nocturnal dipping was defined as a decrease in the systolic and/or diastolic blood pressure during the night ≥10% relative to daytime blood pressure.

**References:**

1. Lurbe E, Agabiti-Rosei E, Cruickshank JK, Dominiczak A, Erdine S, Hirth A, et al. 2016 European Society of Hypertension guidelines for the management of high blood pressure in children and adolescents. J Hypertens. 2016;34(10):1887-920.

2. Meng L, Zhao D, Pan Y, Ding W, Wei Q, Li H, et al. Validation of Omron HBP-1300 professional blood pressure monitor based on auscultation in children and adults. BMC Cardiovasc Disord. 2016;16:9.

3. Neuhauser HK, Thamm M, Ellert U, Hense HW, Rosario AS. Blood pressure percentiles by age and height from nonoverweight children and adolescents in Germany. Pediatrics. 2011;127(4):e978-88.

4. Wühl E, Witte K, Soergel M, Mehls O, Schaefer F. Distribution of 24-h ambulatory blood pressure in children: normalized reference values and role of body dimensions. J Hypertens. 2002;20(10):1995-2007.

**SUPPLEMENTARY 2**

**Demographic and laboratory data of obese children according to gender**

|  | **Obese boys** | **Obese girls** | ***p* value** |
| --- | --- | --- | --- |
| **Number (male/female)** | 57 | 63 |  |
| **Age (years)** | 12.9 ± 3.1 | 13.1 ± 2.9 | 0.790 |
| **Gestation week (weeks)** | 39.6 ± 1.6 | 39.5 ± 1.3 | 0.191 |
| **Birth height (cm)** | 50.5 ± 2.4 | 49.8 ± 2.4 | 0.297 |
| **Birth weight (kg)** | 3401.5 ± 625.8 | 3246.7 ± 588.7 | 0.150 |
| **Height-SDS** | 0.63 ± 1.09 | 0.60 ± 0.97 | 0.888 |
| **BMI-SDS** | 5.63 ± 2.75 | 5.59 ± 2.15 | 0.590 |
| **Day SBP-SDS** | 0.445 ± 1.159 | 0.328 ± 1.612 | 0.654 |
| **Day DBP-SDS** | 0.020 ± 1.644 | -0.298 ± 1.315 | 0.250 |
| **25(OH)D (ng/mL)** | 28.1 ± 8.2 | 27.4 ± 8.9 | 0.337 |
| **Serotonin (ng/mL)** | 143.7 ± 81.5 | 161.9 ± 78.5 | 0.905 |
| **Irisin (ng/mL)** | 8.5 ± 4.0 | 9.4 ± 4.4 | 0.332 |
| **T-Chol (mmol/L)** | 4.27 ± 1.0 | 4.05 ± 0.93 | 0.287 |
| **TAG (mmol/L)** | 1.29 ± 0.54 | 1.28 ± 0.52 | 0.959 |
| **LDL-C (mmol/L)** | 1.08 ± 0.20 | 1.15 ± 0.25 | 0.149 |
| **HDL-C (mmol/L)** | 2.82 ± 0.88 | 2.51 ± 0.78 | 0.086 |
| **Glu (mmol/L)** | 4.42 ± 0.95 | 4.39 ± 0.78 | 0.823 |
| **Insulin (mU/L)** | 25.9 ± 14.0 | 30.9 ± 18.7 | 0.093 |
| **HOMA** | 5.005 ± 2.873 | 5.991 ± 4.589 | 0.144 |
